# Evaluation of a mosquito home system for controlling *Aedes aegypti*

**DOI:** 10.1186/s13071-021-04918-9

**Published:** 2021-08-18

**Authors:** Ahmad Mohiddin Mohd Ngesom, Anis Ahmad Razi, Nur Syahirah Azizan, Nazni Wasi Ahmad, Asmalia Md Lasim, Yanfeng Liang, David Greenhalgh, Jasmine Chia Siew Min, Mazrura Sahani, Rozita Hod, Hidayatulfathi Othman

**Affiliations:** 1 Center for Toxicology and Health Risk, Faculty of Health Sciences, Universiti Kebangsaan Malaysia, 50300 Kuala Lumpur, Federal Territory of Kuala Lumpur, Malaysia; 2 Medical Entomology Unit, Infectious Disease Research Centre, Institute for Medical Research, National Institute of Health, Ministry of Health, 40170 Shah Alam, Selangor Malaysia; 3 Phytochemistry Unit, Herbal Medicine Research Centre (HMRC), Institute for Medical Research, National Institute of Health, Ministry of Health, 40170 Shah Alam, Selangor Malaysia; 4 Department of Mathematics and Statistics, University of Strathclyde, Glasgow, G1 1XH UK; 5 Department of Biomedical Science, Faculty of Medicine and Health Science, Universiti Putra Malaysia, 43400 Serdang, Selangor Malaysia; 6 Department of Community Health, Faculty of Medicine, Universiti Kebangsaan Malaysia, 50600 Cheras, Kuala Lumpur, Malaysia

**Keywords:** Autodissemination, Horizontal transfer, Inhibition emergences, Insect growth regulators, Vector control management

## Abstract

**Background:**

Dengue is a significant public health issue that is caused by *Aedes* spp. mosquitoes. The current vector control methods are unable to effectively reduce *Aedes* populations and thus fail to decrease dengue transmission. Hence, there is an urgent need for new tools and strategies to reduce dengue transmission in a wide range of settings. In this study, the Mosquito Home System (MHS) and Mosquito Home Aqua (MHAQ) formulations were assessed as commercial autodissemination traps in laboratory and small-scale field trials.

**Method:**

Multiple series of laboratory and small-scale field trials were performed to assess the efficacy of MHS and MHAQ exposed to *Ae. aegypti*. In the laboratory trials, various parameters such as fecundity, fertility, wing size, oviposition preferences, residual effects, and MHAQ transference to other containers through controlled experiments were tested. For small-scale field trials, the efficacy of the MHS and MHAQ approaches was determined to ascertain whether wild mosquitoes could transfer the MHAQ formulation from MHS stations to ovitraps.

**Results:**

The data revealed that *Ae. aegypti* was highly susceptible to low concentrations of MHAQ formulations and had a residual effect of up to 3 months, with MHAQ exposure affecting fecundity, fertility, and mosquito wing size. In the oviposition studies, gravid females strongly preferred the hay infusion compared to tap water and MHAQ during egg-laying in the laboratory. Nevertheless, the use of commercial MHAQ by MHS was highly attractive in field settings compared to conventional ovitraps among local *Aedes* spp. mosquitoes. In addition, MHAQ horizontal transfer activities in the laboratory and small-scale field trials were demonstrated through larval bioassays. These findings demonstrated the potential of MHAQ to be transferred to new containers in each study site*.*

**Conclusion:**

This study provided proof of principle for the autodissemination of MHAQ. Through further refinement, this technique and device could become an effective oviposition trap and offer an alternative preventive tool for vector control management.

**Graphical abstract:**

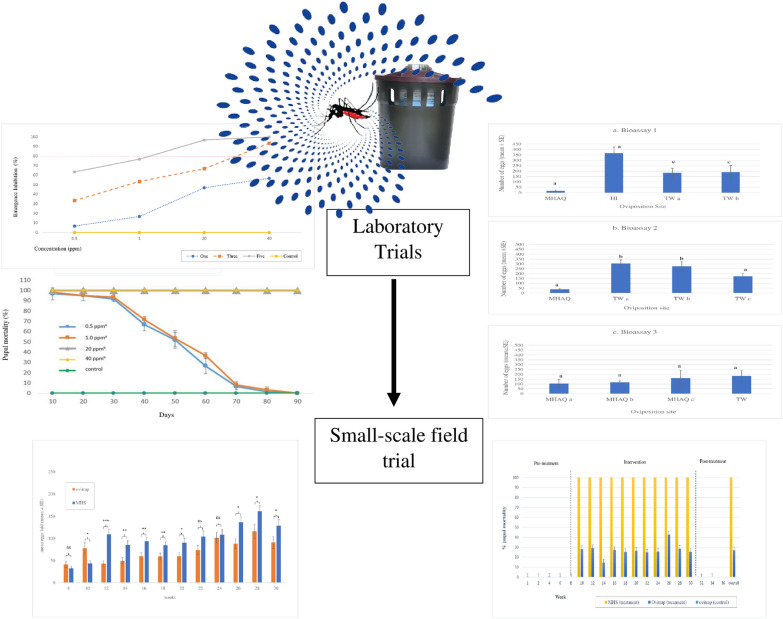

## Background

*Aedes aegypti* and *Aedes albopictus* are both primary dengue vectors found in Malaysia [1], which ranks third in the number of dengue cases reported among countries in the Western Pacific Region [2]. Dengue can be transmitted through the bites of female *Aedes* spp. mosquitoes, with *Ae. aegypti* and *Ae. albopictus* most efficiently adapted to human dwelling areas with artificial container habitats that have a large amount of food.

Although the first licensed dengue vaccine named Dengvaxia was developed against all dengue serotypes [3], the implementation of Dengvaxia was limited to subnational public health programs in Brazil and the Philippines [4]. It offers limited efficacy and can increase the severity of dengue, especially against serotypes 1 and 2 [5, 6]. Therefore, the best prevention strategy is to control the populations of *Aedes* mosquitoes and minimize the presence of mosquitoes [7, 8].

The control measures against *Aedes* spp. populations are based on the recommendations of the World Health Organization (WHO), including implementing source reduction strategies, larvicides, biological controls, lethal oviposition trap, and insecticides, which are used intensively during dengue outbreaks. In Malaysia, thermal fogging and ultra-low volume sprays are the primary interventions when dengue cases are reported [9]. However, uncontrolled use of insecticides during epidemics resulted in increased resistance among mosquitoes to major pesticide classes such as pyrethroids, organophosphates, and carbamate classes [10–13].

Pyriproxyfen is a broad-spectrum insect growth regulator that inhibits the emergence of mosquitoes [14] and is categorized as an eco-friendly pesticide, whereby its application does not remain in water and soil, and its biological degradation cycle serves as a carbon source for other microorganisms [15, 16]. In addition, pyriproxyfen has also been approved for use in drinking water at the recommended application rate [17]. Pyriproxyfen possesses an unexploited potential as a larvicide against mosquitoes, with the autodissemination of pyriproxyfen being suggested by researchers as a novel form of vector control [18–20].

Autodissemination is a very promising and attractive concept that exploits the behavior of female mosquitoes to transfer small particles of insecticides to other cryptic sites [21] and thus prevents the emergence of immature larvae [22]. This was initially demonstrated in the laboratory [23] and subsequently validated by other researchers [24]. Recently, Abad-Franch et al. discovered that mosquitoes could efficiently transfer pyriproxyfen to artificial sites with 100% coverage of dwelling sites within 50 ha, which generated more than a ten-fold increase in pupal mortality and, as a result, led to a decrease in adult emergence [25]. Studies using autodissemination techniques conducted in Manacapuru, a city in Brazil with 60,000 inhabitants, have shown a reduction in the *Ae. aegypti* and *Culex* spp. populations [26]. Further research demonstrated that female mosquitoes could be contaminated, leading to the transfer of pyriproxyfen to other oviposition sites and significantly inhibited adult emergence in laboratory settings and under field conditions [18].

The Mosquito Home System (MHS) is the first commercial trap in Malaysia to adopt the autodissemination concept using the Mosquito Home Aqua (MHAQ) solution. The trap is made of inexpensive polyethylene and operates without electricity or any additional extensions. Its design is based on a gravity-fed watering system that effectively dispenses a trap solution for up to 2 months. Female mosquitoes are exposed to the solution inside the traps by tarsal contact during oviposition, and the insecticide is transferred to other oviposition sites. Over the years, several studies have been performed on MHS and MHAQ and have yielded promising results, although they have been poorly documented [27]. Thus, this study aimed to investigate the efficacy of autodissemination approaches against *Ae. aegypti* in laboratory settings using the MHS devices and MHAQ formulations. In small-scale field evaluations, the efficacy of the MHS and MHAQ approaches were evaluated to ascertain whether wild mosquitoes were able to transfer the MHAQ formulation from MHS stations to the ovitraps. It was anticipated that MHS and MHAQ may have a significant impact on *Ae. aegypti* and potentially emerge as a vector control tool.

## Methods

### Mosquitoes

A susceptible strain of *Ae. aegypti* known as the Institute for Medical Research IMR strain, which originated in Selangor, was used in all experiments in this study and was free from any insecticide exposure. It was maintained in colonies for more than F1000 generations in the insectarium of IMR, Kuala Lumpur. The mosquitoes were reared in the Laboratory of Medical Entomology, Universiti Kebangsaan Malaysia at 26 ± 4 °C and 60 ± 20% relative humidity (RH). Larvae were fed TetraMin® fish food, and cotton soaked with 10% sucrose was fed to adult mosquitoes as nourishment. Female mosquitoes obtained from the F_0_ generation fed on guinea pig (*Cavia porcellus*) blood, and their F_1_ and F_2_ progenies were used in the present study.

### Pyriproxyfen

MHAQ is a commercial solution containing 0.004% pyriproxyfen and is used in conjunction with the MHS device (One Team Networks, Sdn. Bhd., Malaysia). Full information about the formulation cannot be disclosed as it is a trade secret of the company.

### Mosquito Home System as autodissemination station

**Fig. 1:**
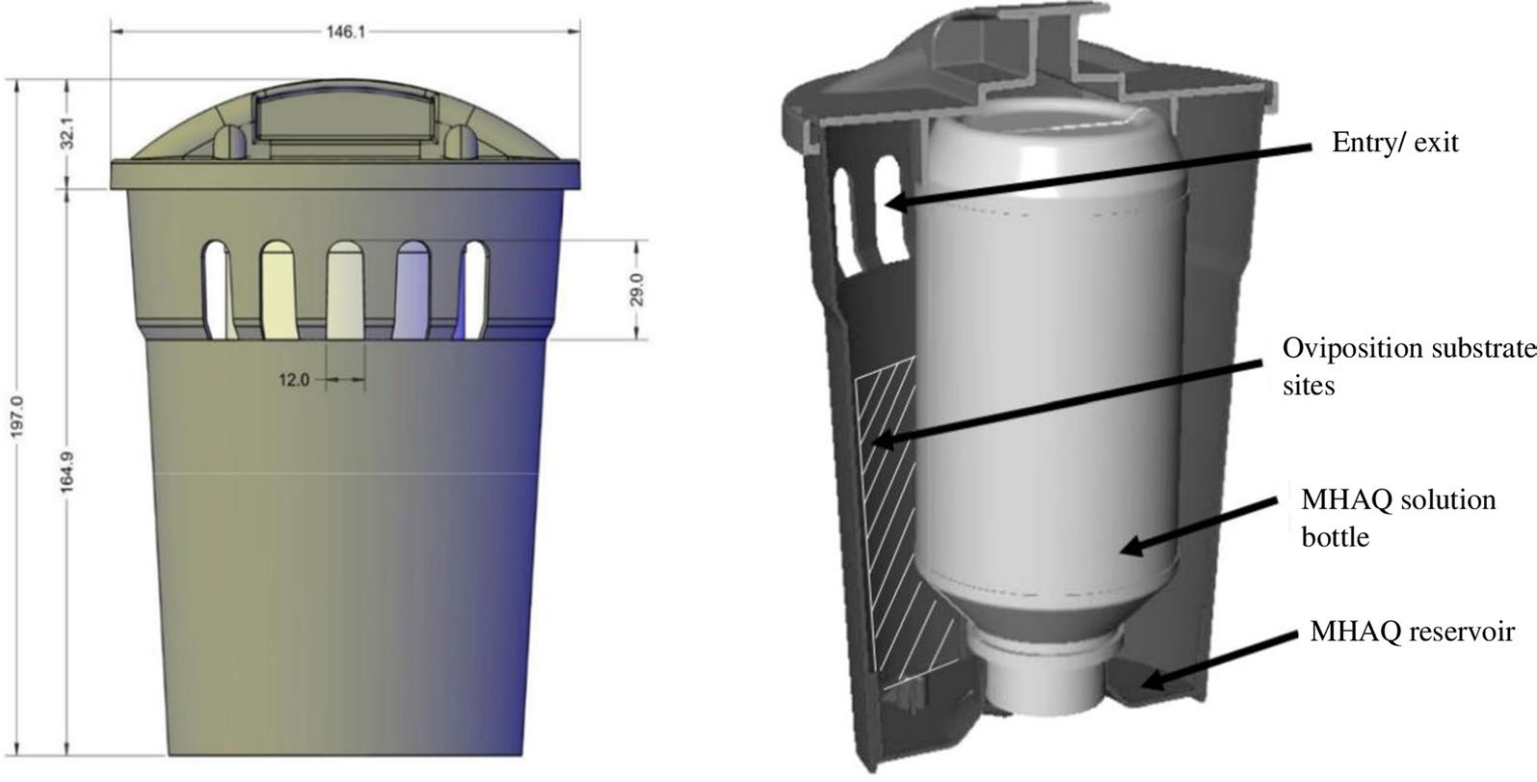
Cross section and schematic image of the MHS and MHAQ solution bottle

The MHS trap (Fig. 1) is a cylinder-shaped black polyethylene container that is 19.7 cm high and 14.6 cm wide. The MHAQ formulation attracts mosquitoes to enter and leave the trap through a row of holes located at the top of the station. The inside of the trap has a black surface with a bottle screw adapter at the bottom. The MHAQ solution bottle can be easily screwed to the base, which is convenient when the solution bottle needs replacing. The design utilizes gravity flow to provide a continuous flow of the solution into the reservoir, with the flow ceasing once the reservoir is filled, ensuring the availability of the formulation for up to 2 months. Traps were lined with paper towels as an oviposition substrate for *Aedes* spp. mosquitoes, and mosquitoes that were exposed to the solution became contaminated with MHAQ when they entered the trap to oviposit on the paper towel and remained contaminated when they exited to find other containers.

### Evaluation of dose response to MHAQ

Larval bioassays were performed using the standard WHO larval bioassay [28]. Eight different concentrations of MHAQ (0.02 ppm to 1.28 ppm) were used, resulting in a range of larval mortality from 0 to 100%. A total of 100 *Ae. aegypti* third-instar larvae were exposed to each of the concentrations, and 50 larvae were exposed to a control containing distilled water with no insecticide. Each cup containing a specific concentration of MHAQ was made by mixing 99 ml of distilled water and 1 ml of insecticide according to the desired concentration. The control treatment consisted of 99 ml of distilled water and 1 ml acetone, and each concentration was replicated four times. Pupal mortality was recorded after an exposure of 24 h, and the lethal concentration (LC_50/90_) was calculated.

To obtain the emergence inhibition (EI), preliminary tests were conducted by exposing the test larvae to a wide range of concentrations. Based on these results, nine different concentrations (0.07 ppb–0.70 ppb) of MHAQ were used, yielding between 5 and 95% in the range of EI. The bioassays were performed using 20 *Ae. aegypti* third-instar larvae for each concentration. The larvae were placed into a 250-ml paper cup containing 99 ml distilled water and 1 ml insecticide and then placed inside a mosquito cage. The control treatment contained 99 ml distilled water and 1 ml acetone, and five replicates were tested for each concentration. Due to the delayed action of pyriproxyfen, larval and pupal mortalities were assessed every day until all individuals had either emerged or died in the control group. Throughout the study, larvae were provided with TetraMin® fish food at 100 mg/liter until pupae emerged [29, 30]. For each bioassay, the temperature was maintained at 26 ± 4 °C with 60 ± 20% RH and a 12-h light: 12-h dark photoperiod.

### Transfer of MHAQ to untreated containers

To demonstrate the transfer of MHAQ to another container, binary choice tests were performed in a small mosquito cage (L: 30 × W: 30 × H: 30 cm) under laboratory conditions of 26 ± 2 °C and 60 ± 20% RH, based on the studies by Chism & Apperson [24] and Sihuincha et al*.* [31]. A binary choice test [24] was performed with each cage holding a batch of gravid females (1–2 weeks old, 4 days post-blood-feeding). Two ovitraps (250 ml capacity, 7.2 cm diameter, 9 cm height) were placed by simple randomization in diagonal corners of each cage. The treatment cage contained two ovitraps: one with an oviposition strip treated with MHAQ and the other with an untreated filter paper in tap water only. The control cage also contained two ovitraps, both lined with untreated filter paper and filled with 100 ml tap water. The MHAQ was prepared as an emulsifiable form to be released effectively during oviposition of the mosquito [20]. Cotton balls soaked with 10% sucrose were supplied in each cage.

Gravid female mosquitoes from the same batch were given a dual choice [24] between the particular concentration of MHAQ and tap water under the following conditions: (1) one gravid with 0.5 ppm, (2) one gravid with 10 ppm, (3) one gravid with 20 ppm, (4) one gravid with 40 ppm, (5) three gravid with 0.5 ppm, (6) three gravid with 10 ppm, (7) three gravid with 20 ppm, (8) three gravid with 40 ppm, (9) five gravid with 0.5 ppm, (10) five gravid with 10 ppm, (11) five gravid with 20 ppm, and (12) five gravid with 40 ppm of MHAQ. To ensure that ovitrap placement did not influence oviposition choice, the ovitrap was rotated clockwise over time in each replicate of each experiment. Each treatment was replicated five times, and the complete assays were repeated three times. The mosquitoes were left to lay eggs, and all the samples and equipment were discarded after 72 h. Twenty larvae were introduced into water samples collected from ovitraps filled with tap water (treatment cage and control cage) and monitored daily until all the larvae and pupae in the control ovitraps had either died or emerged as adults. Successful MHAQ contamination and transferability were evaluated by comparing the differences in larvae/pupal mortality and EI of each ovitrap between treatment and control experiments.

### Oviposition preferences of *Aedes aegypti* to MHAQ in competition with water

**Fig. 2:**
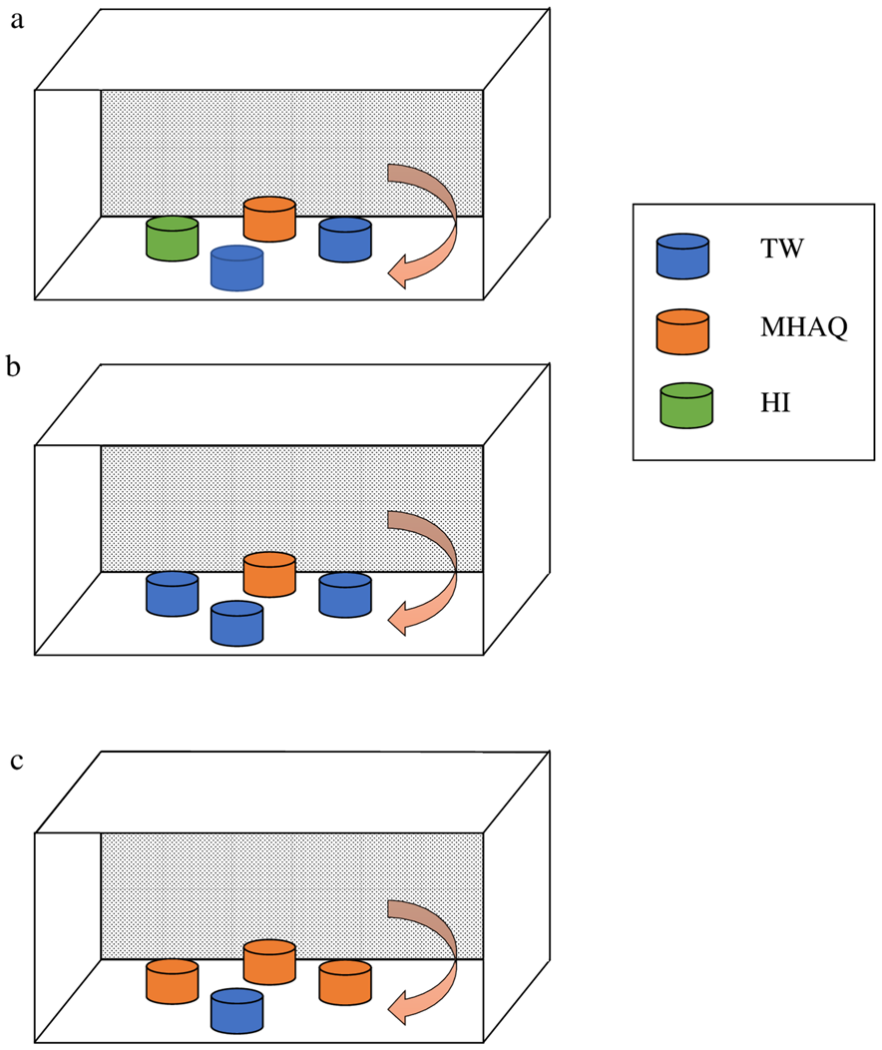
Oviposition bioassay design. The purple curved arrows indicate the changes in direction of the oviposition cup positions in the cages. **a** Four oviposition sites with: (i) Mosquito Home Aqua (MHAQ), (ii) hay infusion (HI), (iii) tap water a (TW a), and (iv) tap water b (TW b). **b** Four ovipositions contained (i) MHAQ, (ii) TW a, (iii) TW b, and (iv) TW c. **c** Four ovipositions contained (i) MHAQ a, (ii) MHAQ b, (iii) MHAQ c, and TW

The oviposition site selection bioassay procedures have been slightly modified based on previous reports [32, 33]. The attraction of *Ae. aegypti* to commercial MHAQ solutions in the presence of multiple oviposition sites was determined using a cage containing 60 females (3–5 days non-blood-fed) and 15 males (2–5 days old), and blood meal was offered after 30 min of combining. After 3 days of blood-feeding, 15 females were randomly transferred to a new cage containing four oviposition cups filled with a Whatman® No. 1 filter paper (Merck Millipore, USA) and 200 ml of the following solutions: (i) MHAQ solution—MHAQ, (ii) Hay Infusion—HI, (iii) tap water a—TW a, and (iv) tap water b—TW b, respectively (Fig. 2a).

In a second experiment, the preference of female mosquitoes to oviposit in small water sources was determined by exposing 15 females to the following solutions: (i) MHAQ, (ii) TW a, (iii) TW b, and (iv) TW c (Fig. 2b). In addition, the preference of mosquitoes to oviposit in various containers was also assessed in the third experiment using the MHAQ a, MHAQ b, MHAQ c, and TW a solutions (Fig. 2c). Every oviposition site containing MHAQ solution and tap water in each experiment was replicated four times, and the position of the cup was randomized (Fig. 2). A new batch of 15 females was used in each experimental replicate. In all three experiments, the mosquitoes were allowed to lay eggs for 3 days on the Whatman® No. 1 filter paper, and their egg deposition was assessed through examination of the filter paper.

### Residual larvicide activity of MHAQ

To assess the residual larvicidal activity of MHAQ, four different concentrations (0.5 ppm, 1.0 ppm, 20 ppm, and 40 ppm) were evaluated in laboratory settings and covered with a net to prevent ovipositing of any mosquitoes. Five beakers (15 cm diameter × 18 cm height) were placed approximately 30 cm from each other, with four beakers filled with 2.0 l of different MHAQ solution concentrations and the remaining beaker with tap water as a control. These bioassays were performed in replicates of three for every concentration and control. Batches of 20 larvae were released on day 0, with the replacement of larvae carried out every 10 days via the introduction of new larvae batches, and observation was conducted for 90 days. Every time larvae were replaced, all the larvae and pupae were counted and removed and the surviving larvae transferred into paper cups for further observations. Larvae were supplied with TetraMin® fish food, and experiments were carried out at 26 ± 4 °C and 60 ± 20% RH with a 12:12 day:night photoperiod.

### Effect of MHAQ on fecundity and fertility

Colonies of *Ae. aegypti* were subjected to a sublethal dose LC_50_ [34] (where 50% of the mosquitoes could still be alive and capable of producing first-generation progeny) of MHAQ following the WHO larval bioassay method. After 24-h exposure, the surviving larvae were transferred and allowed to emerge in emergence cages. The larvae were fed TetraMin® fish food, while the adult mosquitoes were fed 10% sucrose solution. After 3 to 5 days, 30 male and female mosquitoes were transferred into oviposition cages for cohabitation, and 30 min after combining, the mosquitoes were provided with guinea pig blood for 2 h. Fully engorged mosquitoes were then transferred to a new cage and supplied with a 10% sucrose solution. Three days of blood meal was provided; 15 females were transferred to individual plastic cups that were covered with a net and lined internally with a Whatman® No. 1 filter paper as an oviposition substrate. The females were allowed to oviposit their eggs for 5 days, and the number of eggs was counted daily [18]. Eggs collected during the fecundity test were used in the fertility test by immersing the filter paper with the eggs into the tap water in the culture trays. The number of larvae that hatched was monitored and recorded, and the experiments were carried out at 26 ± 4 °C and 60 ± 20% RH with a 12:12 day:night photoperiod.

### Adult wing length

It is known that the adult wing length correlates with the fecundity and body size of mosquitoes. After the adults emerged from the fertility studies, a total of 30 *Ae. aegypti* mosquitoes (males and females) were anesthetized and killed. All the right wings were removed, and the length of the wings was measured from the axial vein to the radius 1 (R1) vein using a stereomicroscope (100×).

### Small-scale field trials

According to WHO guidelines, new products that have shown promising results in laboratory studies (Phase I) should be evaluated under Phase II. At this point, a proof of concept was required, i.e. the MHAQ could be transferred to other containers by wild mosquitoes and subsequently the larvae could be killed. The study sites were located in the Petaling District, Shah Alam, Selangor, and two similarly isolated areas were selected: Dataran Automobil as the treatment area (19°57′05″ S, 43°76′88″) and Seksyen 16 as the control area (19°57′05″ S, 43°76′88″). The distance between the treatment and the control site was approximately 1.8 km, and both areas have been declared dengue ‘hotspots,’ with continuous dengue cases being reported, and shared a similar housing structure with good access to sanitation. Dataran Automobil was selected as a treatment area because it is more vulnerable than Seksyen 16 and is closer to our workstation in Shah Alam.

The trial consisted of a 2-month pre-treatment period (November–December 2017), 6 months of treatment, and 1 month of post-treatment (January 2018–July 2018). In the treatment and control areas, 48 ovitraps were placed in each area, with a total of 96 ovitraps [20, 32]. All the ovitraps were randomly positioned in the potential container, such as under roofs, near vegetation sheds, water supplies, and near human activities areas. Each ovitrap cup was individually coded with labels to ensure it was placed at the same location in each sampling round and any missing ovitrap or paddle was replaced with a new one.

During the treatment phase, from the third month onwards, a total of 48 MHS stations were deployed at a distance of 1 to 10 m from the nearest ovitrap across the treatment site from January 2018 to June 2018. The stations were only deployed at the treatment site and serviced on a fortnightly basis to ensure that they were completely operational, that there was sufficient volume of the solution, and that any clogged devices were removed. The stations were then examined and the remaining solutions and paper substrates were collected and brought back to the laboratory for larval bioassay assessment.

The ovitraps and MHS were taken to the laboratory separately, and the contents of each MHS and ovitrap, such as water, paddles, and paper substrates, were transferred to an enamel pan to facilitate the observation of mosquito juveniles and attached eggs. All larvae were identified [35], and total intact eggs (hatched or unhatched) and live larvae were counted and recorded.

The impact of treatment using the MHS to deliver MHAQ to other ovitraps was determined using larval bioassays as defined in the WHO guidelines. In the case of ovitrap containers, water samples from treatment and control areas were tested at three different time points: pre-intervention, intervention, and post-intervention. In addition, although water samples from the MHS were available only during the treatment period, these samples were also tested. Larval bioassays were performed using late third-instar larvae of the *Ae. aegypti*. Control treatments were treated with 199 ml tap water and 1 ml of acetone while three cups were set up using tap water and 20 larvae per bioassay as negative controls. Mortality was recorded every 24 h until adult emergence, and larvae were also provided with food daily. In certain samples, pyriproxyfen contamination increased the larval development time from a typical 8 to 9 days up to 14 days, resulting in pupae death. The experiments were conducted at 26 ± 2 °C, 60 ± 20% RH, and preferably a photoperiod of 12 h light followed by 12 h dark.

### Statistical analysis

All statistical analyses were conducted using the Statistical Package for the Social Science (SPSS) version 23. In each assay, the data sets were tested for normality distribution using the Shapiro-Wilk test. Prior to analysis, the log_10_ values of the data were obtained. If the data were normally distributed, a parametric test was performed, followed by a Tukey post hoc test. However, if the data were not normally distributed, a non-parametric test was applied [36].

All data for each treatment bioassays were presented as a mean ± SE unless specified otherwise. For the larvae bioassay test, data were analyzed using the probit analysis. The EI was calculated using the following formula [37]:

$${\text{Percentage of inhibition of emergence }} = { 1}00 \, {-}{ 1}00\left( {T/C} \right)$$

where *T* is the number of emergence in treated containers, and *C* is the number of emergence in control containers. Control mortality > 5% was corrected using Abbott’s formula [38].

Mortality data for the larval bioassay were recorded and presented as a percentage, with all data calculated using the log-probit analysis. Results were presented as LC_50_ (lethal concentration in ppm for 50% death) and LC_90_ (lethal concentration in ppm which caused 90% mortality).

The effective reduction (ER) percentage was calculated as:

$${\text{ER}}\% \, = \, \left[ {{\text{NC}} - {\text{NT}}/{\text{NC}}} \right] \, \times { 1}00$$

where NC is the number of eggs in the control group and NT is the number of eggs in the treatment group. Student’s t-test was used to compare fertility, fecundity, and wing length of *Ae. aegypti* between the control and treated groups. A Mann-Whitney test was performed when normality was not met.

The comparison of oviposition attractants was performed using a one-way ANOVA test followed by a Tukey post hoc test. The impact of the oviposition site with MHAQ, HI, and TW was assessed with a one-way ANOVA test, followed by a Tukey post hoc test where necessary. All the results of the analysis with *p* < 0.05 were considered statistically significant. For the residual activity trials, analysis of the concentration effects, pupal mortality, and their interactions was performed using two-way repeated measures ANOVA and the Greenhouse-Geisser correction. The transfer activities of MHAQ in the laboratory and field trials were analyzed for each concentration for successful adult emergence using a one-way ANOVA with a Tukey post hoc test between the means (*p* < 0.05).

## Results

### Susceptibility of *Ae. aegypti* to MHAQ

The response dose range was established based on the third-instar *Ae. aegypti* larvae. Mortality of the *Ae. aegypti* was recorded approximately 24 h after exposure in the laboratory bioassay, and it was observed that MHAQ effectively killed the *Ae. aegypti* larvae at 24 h exposure with a LC_50_ of 0.0903 ppm (95% CL = 0.0827–0.0986) and LC_90_ of 0.237 ppm (95% CL = 0.237–0.286). Meanwhile, the EI_50_ and EI_90_ of *Ae. aegypti* exposed with MHAQ were 0.323 ppb (95% CL = 0.065–0.89) and 0.102 ppb (95% CL = 0.059–0.141), respectively.

### Transfer of MHAQ from treated to untreated ovitraps

**Fig. 3:**
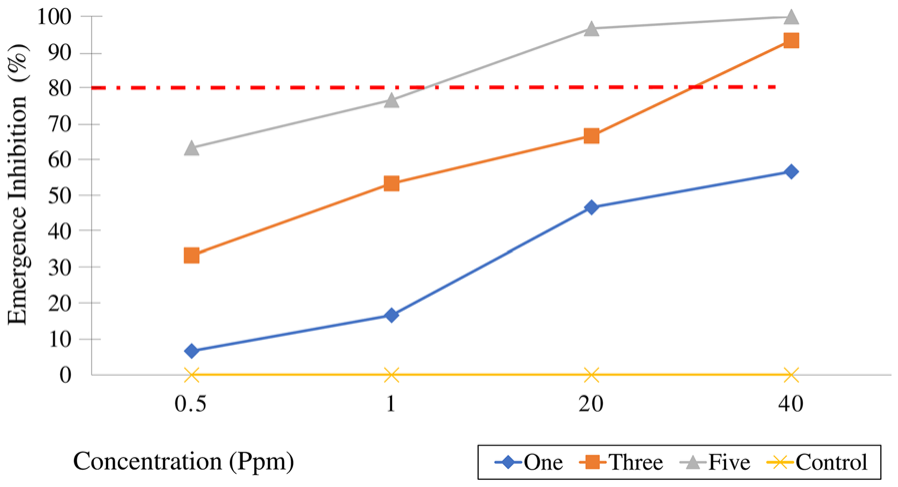
*Aedes aegypti* as transportation for MHAQ transfer: Emergence inhibition over the MHAQ different concentrations for *Ae. aegypti* in untreated water. The red dashed line shows the threshold value of 80% above which the insecticide is considered effective

The ability of one, three, and five mosquitoes to transfer MHAQ to the untreated containers was determined, and larvae were significantly prevented from developing into adults compared to the control groups (ANOVA, *F*_(6, 24)_ = 3.97, *p* < 0.007). For treatment groups, the five-mosquito group transferred the most MHAQ and had the highest EI compared to the other groups. However, there was no significant difference in the groups of three and five females at 40 ppm of MHAQ with an EI% between 93.3 and100% (Fig. 3). Table 1 shows the exposure of different numbers of mosquitoes at oviposition sites treated with different concentrations of pyriproxyfen. The results revealed that there was no significant difference between the number of eggs laid in the treated and untreated water by a different number of mosquitoes. It was evident that even at high concentrations, MHAQ did not affect the number of eggs that had been laid.

**Table. 1:** Mean number (± SE) of eggs laid in treated and untreated ovitraps with different ranges of MHAQ concentration and number of mosquitoes

No. of mosquitoes	Concentration (ppm)	Mean no. of eggs laid		*P-*value
		Treated	Untreated	
One	0.5	16.00 ± 2.31^a^	9.00 ± 2.00^a^	1.00
	1.0	6.33 ± 2.19^a^	5.51 ± 3.18^a^	0.38
	20	8.67 ± 2.03^a^	9.00 ± 2.65^a^	0.61
	40	12.67 ± 2.85^a^	7.00 ± 3.61^a^	0.64
Three	0.5	57.00 ± 11.14^a^	47.00 ± 10.44^a^	0.78
	1.0	42.67 ± 10.33^a^	16.67 ± 5.24^a^	0.28
	20	31.33 ± 7.75^a^	23.00 ± 4.16^a^	0.21
	40	48.00 ± 11.72^a^	18.33 ± 6.69^a^	0.38
Five	0.5	67.67 ± 32.35^a^	40.33 ± 7.13^a^	0.12
	1.0	114.33 ± 32.85^a^	62.67 ± 24.91^a^	0.04
	20	66.67 ± 16.83^a^	47.67 ± 19.92^a^	0.93
	40	78.00 ± 21.20^a^	51.33 ± 8.65^a^	0.11

Groups in the same row that shows a different letter are significantly different (p < 0.05)

### *Aedes aegypti* oviposition activities to MHAQ in competition with water

**Fig. 4:**
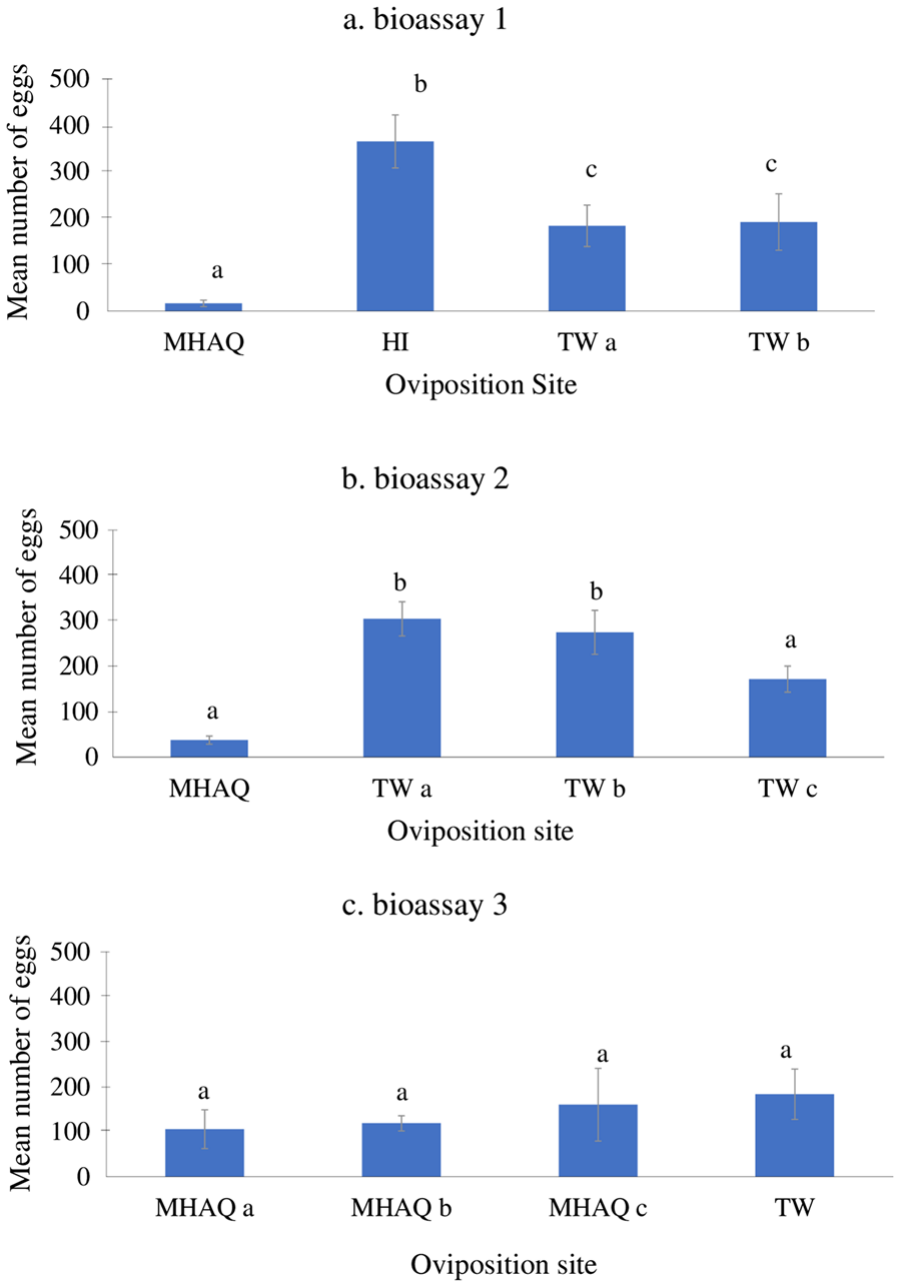
Response of gravid female *Aedes aegypti* when given a choice to oviposit in four cups containing MHAQ at different concentrations with water. **a** Oviposition sites: (i) MHAQ, (ii) HI, (iii) TW a, and (iv) TW b. **b** Oviposition sites: (i) MHAQ, (ii) TW a, (iii) TW b, and (iv) TW c. **c** Oviposition sites: (i) MHAQ a, MHAQ b, MHAQ c, and TW. Means sharing the same letter are not statistically different from one another (*p* > 0.05)

Three different types of bioassays were used to observe the oviposition preference of *Ae. aegypti* between MHAQ. For the first bioassay, oviposition sites were divided into either MHAQ, HI, TW a, or TW b. Gravid *Ae. aegypti* females laid their eggs in all the cups, and a total of 2107 eggs were recorded. The maximum mean number of eggs laid was in the HI (365.00 ± 57.14), followed by TW b (191.75 ± 60.72), TW a (183.50 ± 44.58) and MHAQ (16.75 ± 6.93). There was a significant difference between the mean number of eggs in the MHAQ with HI and TW (ANOVA, *F*_(3,12)_ = 13.003, *p* = 0.0004). However, no significant difference was observed between TW a and TW b in this assay (*p* > 0.05) (Fig. 4a).

For the second bioassay, a comparison among four cups (three TW and one MHAQ) was conducted to evaluate the oviposition site preference of gravid *Ae. aegypti*, with eggs also laid in all four cups. However, only 4.2% of eggs were deposited in a cup containing MHAQ (MA = 37.5 ± 8.93) while 95.8% were deposited in the cups filled with tap water (TW a: 304.25 ± 37.82; TW b: 274.5 ± 48.56; TW c: 172.25 ± 28.83) (Fig. 4b). There was a significant difference between the two types (MHAQ and TW) of attractants used for oviposition (ANOVA, *F*_(3,12)_ = 12.359, *p* = 0.001). However, there was no significant difference in the mean number of eggs collected in the TW a and TW c (*p* > 0.05).

In the third bioassay, three cups were filled with MHAQ a, MHAQ b, and MHAQ c and one with TW, with eggs oviposited in all the cups. A total of 2269 eggs were laid in all the cups, with 68% of the eggs in the cups baited with MHAQ solutions and 32% of the eggs in the cups baited with TW, resulting in the highest number of eggs laid in the TW cups. Moreover, there was no significant difference between the mean number of eggs collected across the treatments (ANOVA, *F*_(3,12)_ = 0.442, *p* = 0.727) (Fig. 4c).

### Residual larvicide activity of MHAQ

**Fig. 5:**
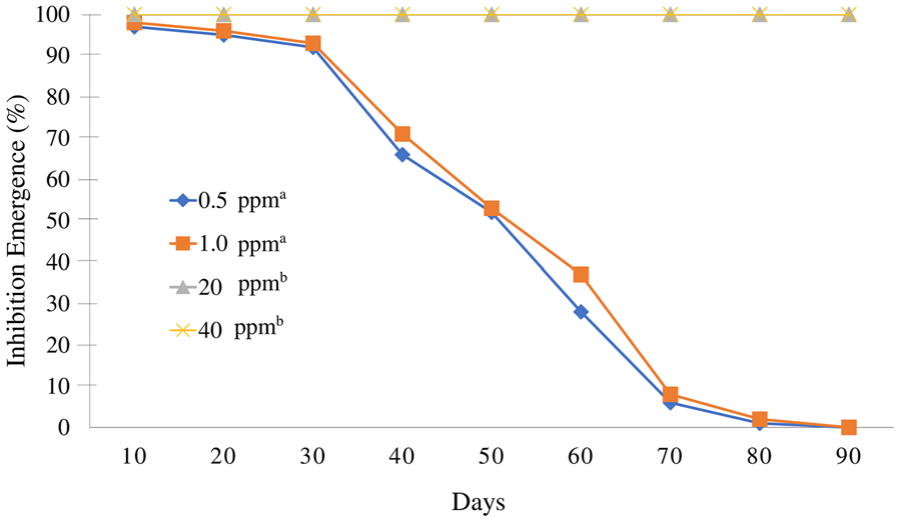
Residual effects of MHAQ at different concentrations on *Ae. aegypti* larvae. The values at each point (every 10 days) represent the mean pupal mortality ± SE (%). The MHAQ concentrations with significant differences are represented by different superscript letters (repeated measures ANOVA; *p* < 0.001 by Tukey post hoc test)

From day 10 to day 90, there was greater efficacy of the MHAQ at the higher concentrations of 40 ppm and 20 ppm. At the lower concentrations (0.5 ppm and 1.0 ppm), there was > 90% mortality until day 30 until a < 80% decrease in pupal mortalities on day 40. There was no pupal mortality on day 90. Based on laboratory trials, the residual activity of the MHAQ achieved > 80% mortality for 30 days and continued for 90 days (Fig. 5). The interaction between the MHAQ concentration and time was statistically significant based on a repeated measure ANOVA and the Greenhouse-Geisser correction on the number of pupal mortalities (time, *F*_(3.77, 94.21)_ = 547.21, *p* < 0.001; treatment, *F*_(4, 25)_ = 7020.44, *p* < 0.001, treatment × time, *F*_(15.07, 94.21)_ = 205.71, *p* < 0.001).

### The effect of MHAQ on fecundity, fertility, and wing length

It was found that the LC_50_ = 0.0903 ppm significantly reduced the number of eggs laid in the treatment group compared to the control group (Student’s *t*-test: *t* = 7.509; df = 18; *p* < 0.05). The mean number of eggs laid by *Ae. aegypti* was 53.70 ± 13.64, compared to 112.80 ± 20.82 in the control group (*p* < 0.05) (Student’s *t-*test for a single sample: *t* = 8.355; df = 10; *P* < 0.001), indicating a 50% reduction in the mean number of eggs laid in the treatment group compared to the control group.

There was also a significant difference (Student’s *t*-test: *t* = − 5.489; df = 18; *p* < 0.05) in the percentage of eggs hatching in the control group (92.80%) and the treatment group that was exposed to a sublethal dose of MHAQ (70.67%). The ER% was ≈ 23.46, which was significantly different from zero (Student's t-test for a single sample: *t* = 5.023; df = 10; *p* < 0.001), suggesting a decrease in the rate (percent) of hatching in the treatment group by 20% compared to the control group. On the other hand, the wing lengths of male mosquitoes that had the sublethal treatment were significantly different from the wing lengths of male mosquitoes in the control group (Mann-Whitney U-test: *U* = 2.5, *Z* = − 6.6635, *p* < 0.0001). The effects were similar for female mosquitoes, where the wing lengths of those that had the sublethal treatment were significantly different from those in the control group (Mann-Whitney U-test: *U* = 0, *Z* = − 6.662, *p* < 0.0001) (Table 2).

**Table. 2:** Effects of MHAQ on fecundity, fertility, and wing length on *Aedes aegypti* mosquitoes

Parameter	Treated	Control
Fecundity		
No. of eggs laid	53.70 ± 13.64^a^	112.80 ± 20.82^b^
ER (%)	52.40	
Fertility		
No. of hatching eggs	70.67 ± 3.64^a^	92.80 ± 1.72^b^
ER (%)	23.46	
Wing length (mm)		
Female	2.23 ± 0.06^a^	2.63 ± 0.05^b^
Male	2.09 ± 0.03^a^	2.19 ± 0.04^b^

Groups in the same row that shows a different letter are significantly different (p < 0.05)

### Efficacy of small-scale field trials

**Fig. 6:**
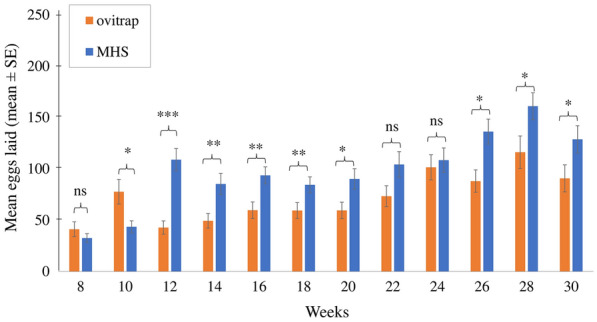
The mean number of eggs (± SE) collected in the ovitraps and MHSs during intervention period at small-scale field trials (*n* = 48). Significant differences are indicated by asterisks (repeated measure—ANOVA, *p < 0.05, **p < 0.01, *** *p* < 0.001, ns = not significant)

Egg collections were carried out from both ovitrap and MHS to determine the preference of the mosquitoes between TW and MHAQ. During the intervention period, MHS with MHAQ formulations were most effective in attracting *Aedes* compared to ovitrap, with a significantly greater collection in MHS (1176.15 ± 57.84) than in ovitrap (858.82 ± 59.87) (ANOVA: *F*_(1, 94)_ = 14.57, *p* < 0.001). After 4 weeks of MHS implementation, an increased number of eggs collected was observed in week 12, and this was consistently observed until the end of week 30. Based on a repeated measure ANOVA and the Greenhouse-Geisser correction, there was a significant difference in the average eggs that were collected between multiple time points (*F*_(7.88, 740.75)_ = 15.24, *p* < 0.001; treatment × time, *F*_(7.88, 740.75)_ = 3.67, *p* < 0.001). In addition, the number of eggs collected fluctuated with some variation due to other environmental factors (Fig. 6).

**Fig. 7:**
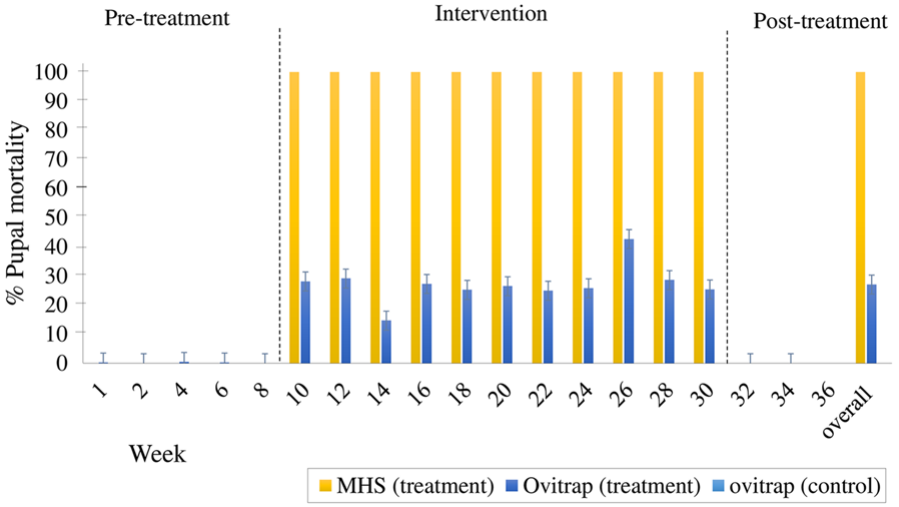
The mean percentage (± SE) of pupal mortality in water samples collected from the MHSs and ovitraps using larval bioassay in the treatment and control areas throughout 36 weeks during 2017–2018

In the small-field trials, autodissemination of MHAQ was detected in the ovitraps. In the ovitrap and MHS, the mortalities of pupas exposed to the water sample during the intervention periods were significantly higher than in the control samples, where pupal mortality in the ovitrap was 28.07 ± 4.42% at week 10, which was consistent during the trials and peaked at 42.63 ± 11.46% at week 26. There was a small decrease in mortality at week 14 (14.61 ± 2.03). After 12 weeks of intervention, the overall mortality in the trials was 27.01 ± 1.8%, and 100% pupal mortality was observed in both the ovitraps and the MHS (Fig. 7).

## Discussion

Dengue fever is a disease caused by mosquitoes that significantly impacts human health. However, existing vector management methods have failed to reduce dengue infections, indicating that these methods may not be sufficient and require improvements. Pyriproxyfen autodissemination is a novel strategy for dispersing pyriproxyfen into oviposition containers through the manipulation of the ‘skip oviposition’ behavior of mosquitoes. The study aimed to determine the effectiveness of MHS using autodissemination strategies based on laboratory and small-scale field studies.

Multiple choice of egg-laying site tests and larval bioassays revealed that *Ae. aegypti* was highly susceptible to low concentrations of MHAQ, which affected the fecundity, fertility, and wing size of the mosquitoes. In this study, MHAQ was more effective in killing *Ae. aegypti* larvae after a 24-h exposure, with the LC_90_ = 0.237 ppm, which was lower compared to a previous study (LC_90_ = 10 ppm) [39]. The EI_50_ rate obtained was 0.323 ppb, which was consistent with other studies that reported low EI_50_ = 0.56 ppb [23], 0.353 ppb, 0.219 ppb [40], and 0.008 ppb [41]. Furthermore, Paul et al*.* [42] showed that pyriproxyfen effects were more toxic against *Ae. aegypti* than other insecticides (methoprene, diafenthiuron, and tebufenozide). The dose-response test result obtained in this study differed from others, which could be due to the mosquito strain selections used in the experiments [43], different pyriproxyfen formulations [44], the material of the test containers [45], and experimental conditions. It was reported by Suman et al*.* [45] that the efficacy of pyriproxyfen was positively associated with the type of substrate used during the study. The LC_50_ value in tires was found to be 50-fold higher compared to glass containers, and it has been suggested that the pyriproxyfen was adsorbed by the substrate used [45]. There have so far been no reports of *Aedes* spp. resistance to pyriproxyfen in Malaysia; thus, it can be used as an alternative insecticide in dengue control programs [46].

In the present study, MHAQ exposure at the selected dose significantly reduced the fecundity, fertility, and wing length of the *Ae. aegypti* mosquitoes. The size of female mosquitoes is important as larger females produce more eggs and contribute more to the mosquito populations. Exposure to pyriproxyfen has been demonstrated to affect the reproductive capacity of *Aedes* spp. through inhibition of vitellogenesis during egg formation [47], which is desirable since the production of a high number of eggs and optimal fertility are two key factors that influence the survival of mosquitoes [48]. Moreover, Suttana et al*.* [49] found a reduction in fertility and fecundity after only 5 min of exposure to pyriproxyfen. On the other hand, although there was no significant difference recorded in the number of eggs laid, Rhyne et al*.* [50] reported a lower hatching rate in treated containers compared to the controls. However, the effects of pyriproxyfen may vary among mosquitoes depending on exposure time, the concentration used, mode of delivery, and other factors [51]. Pyriproxyfen also affects the fertility and fecundity rates of the mosquitoes; however, the mechanism of action remains uncertain and requires further investigation [52–54].

The study conducted on horizontal transfer of MHAQ was crucial as it demonstrated successful autodissemination by the adult *Ae. aegypti* from treated to untreated sites. It would also be useful to target the gravid female mosquitoes at their resting sites as this may increase the amount of insecticide that can be transferred to other oviposition sites. In addition, Chism et al*.* [24] found that the larval mortality in an untreated site could be associated with the number of eggs laid and the residence time in the containers. The longer the time it takes for mosquitoes to lay eggs, the greater their chances of depositing more MHAQ at the untreated sites. The findings of this study provided evidence, in principle, that the MHAQ solution can be significantly transferred from contaminated containers to gravid females, who then transfer it to other oviposition sites. It was found that autodissemination activity increased with the number of female mosquitoes, and the highest EI was observed when five *Ae. aegypti* were introduced to 20 and 40 ppm of MHAQ during the assays. To provide optimal control, the MHAQ needs to be disseminated by a large number of mosquitoes in particular sites, with higher numbers of mosquitoes thought to be able to transfer more chemicals compared to smaller numbers. During these trials, it was presumed that the gravid females would be more contaminated with higher MHAQ concentrations over time during oviposition.

Autodissemination using a powdered formulation of pyriproxyfen is impractical, as its application causes the accumulation of granules at the bottom of the container over some time [55]. Because of this, the MHAQ solution was evaluated concerning its capability in transferring different concentrations of pyriproxyfen to other containers under laboratory conditions. In addition, this was also the first report suggesting the autodissemination approach using commercial MHAQ-treated water to control *Ae. aegypti* populations.

Compared to tap water and MHAQ, more gravid females laid their eggs in hay infusion. MHAQ has an attractant effect, similar to hay infusion, and has been considered the “gold standard” for collecting and attracting mosquitoes [56]. Despite the preference for hay infusion, it was observed that *Ae. aegypti* oviposited eggs in all the treated and untreated ovitraps. Although MHAQ does not have a strong repellent effect, the females would still have an equal preference to lay their eggs in MHAQ and water under natural conditions. In addition, the laboratory test containers were chemically treated, which could be a reason why mosquitoes avoided laying their eggs in the containers and did not spend enough time in the cups. Other studies found that microbial activities in each oviposition bioassay influenced mosquito oviposition preference [57, 58]. Thus, it remained unclear which of the numerous unknown degradants, mechanisms, and sources of the substances reduced the attraction of *Ae. aegypti* to MHAQ with respect to tap water in our study. Physicochemical aspects of the aquatic medium, visual cues, and the likelihood or possibility of adverse effects on larval development have been reported to be major factors affecting egg deposition decisions of female mosquitoes [59]. This study is the first report of MHAQ's effect on the ovipositional activity of mosquitoes, and it is essential to determine which of the chemical structures and compounds in the products affect the oviposition preference.

These findings also revealed the existence of the previously reported “skip oviposition” behavior. Furthermore, the effectiveness of the autodissemination technique highly depends on the *Aedes* mosquitoes acting as a mode of transportation to disseminate insecticide to the other containers [60]. The females distributed their eggs in multiple containers to avoid overcrowding in the same container as well as reduce the possibilities of losing all offspring due to site elimination to ensure the survival of their progeny. This finding was similar to the study conducted by Wong et al*.* [61] in Iquitos, where female mosquitoes laid eggs in all containers, but other parameters (food resources, size, temperature) were strictly assessed. Although gravid female *Ae. aegypti* and *Ae. albopictus* distribute their eggs at many sites, this behavior was not mutually exclusive as it may differ over time depending on the available suitable containers [62].

This study also discovered that MHAQ formulations have repellent properties against *Ae. aegypti* in laboratory settings, leading to a reduction in MHAQ efficacy. These are the first reported trials that assessed the efficacies of the MHS and MHAQ as new products. According to Vontas et al*.* [44], evaluation of all new vector control tools in testing pathway phases must be performed to ensure that one or two primary effects are obtained as a result of laboratory testing. However, there are a few criteria that need to be considered if the new product is to be tested in field trials. One of the criteria was to employ the ‘stop and go’ principle, which states that if a new product assay is not optimum yet but has passed the minimum efficacy threshold in the laboratory, there is merit to proceeding to the next level of testing, i.e. small-scale field evaluation. Furthermore, if the product has multiple properties (e.g. reduce fertility or repellence) and only displayed one action with an acceptable passing “indicative criterion,” it is likely that extra combined effects can be measured at a later phase.

Nevertheless, results obtained from the laboratory assessment must be interpreted with caution as the tests were conducted in a well-controlled environment when making operational field decisions. A variety of biological parameters including type of strain and preference of egg-laying oviposition, as well as environmental parameters such as temperature, humidity, rainfall, and sunlight conditions, can all affect the efficacy of the product [63–65]. Despite laboratory tests revealing the minor repellent impact of MHAQ against mosquitoes, the number of eggs obtained in small-scale field trials was higher than the gold standard (ovitrap). Other parameters such as the chemical transference by mosquitoes and the effect on biological parameters of mosquitoes also indicate a promising outcome. The MHS could potentially be used as an autodissemination station in future control efforts. Moreover, this is the first report of the effect of MHAQ on the ovipositional activity of mosquitoes. It is essential to determine the chemical structures and compounds in the products that affect the oviposition preference.

The beneficial residual impact of MHAQ on *Ae. aegypti* is encouraging and has presented a prolonged action of 22 weeks by killing 100% of the larvae. With respect to the residual larvicidal activity of the commercial MHAQ solutions, concentrations of 20 and 40 ppm were sufficient to produce 100% mortality up to 90 days. Since the MHAQ formulation can be kept inside the MHS for up to 2 months before completely evaporating, MHAQ needed to remain active for at least the 2-month period of the treatments. This slow-release formulation should be able to exhibit residual larvicidal activity to prevent the emergence of mosquitoes and, subsequently, reduce labor costs and spraying activities. Furthermore, WHO considers ≥ 80% mosquito mortality to be an effective insecticide. However, it was noted that the exposure of treated containers under laboratory settings may not reflect actual field conditions as environmental factors such as direct rainfall, organic matter, exposure to sunlight, and water exchange may reduce the efficacy of MHAQ. Therefore, further studies on MHAQ residual activity should be conducted under a wider range of settings.

The findings in the small-scale field trials were consistent with previous studies that demonstrated that MHS was an efficient tool to monitor and trap *Aedes* spp. mosquitoes [66, 67]. The small-scale field trials showed that MHS consuming commercial MHAQ was highly attractive compared to conventional ovitraps among local *Aedes* mosquitoes. Our trials were located in the middle of the city with a high range of mosquito population, with no walls or barriers in place to prevent mosquitoes from migrating into the study area from nearby. These mosquitoes may potentially replace the local mosquito population [68], thus laying their eggs inside the MHSs, ovitrap, or both as their first choice before they reach other container habitats. It is important to address this significant factor, especially in the open field trials [69]. Apart from varying moisture levels, visual and heat cues may also affect mosquito recognition of hosts, in particular environments that produce different results. Thus, further studies are still required on whether the MHAQ is consistently more effective in different settings.

In a field setting, the efficiency of MHAQ dissemination from MHS to ovitrap was demonstrated. The occurrence of MHAQ in the ovitrap was determined, with pupal mortality ranging from 14.6% to 42.6%. It was discovered that no larvae of *Aedes* spp. were present in the MHS stations treated with MHAQ during the MHS implementation period, leading to the hypothesis that the MHAQ formulation killed all of the hatched larvae and demonstrated the effectiveness against *Ae. aegypti* first-instar larvae. Our previous study using residual activity trials found that MHAQ (20 and 40 ppm) had 100% mortality against *Aedes* mosquitoes for up to 3 months, which implied that the 100% mortality observed in the water sample collected from MHS (treatment) in the small-scale field trials was also influenced by the residual effect. Aside from that, the initial concentration of MHAQ was maintained by the replenishment of the formulation every 2 weeks. Semi-field and field trials have demonstrated that *Aedes* spp. can transfer pyriproxyfen from the treated containers to other larval oviposition sites, which have caused a high range of EI rates on the mosquitoes. Suman et al. [70] obtained 50.4% pupal mortality against *Ae. albopictus* with a dissemination range of up to 200 m in residential areas while Lloyd and colleagues also reported autodissemination activities within a range of up to 200 m from the autodissemination vases that can be effectively used for 5 weeks against *Ae. albopictus* larvae [71]. Thus, it is likely that most studies used a large amount of pyriproxyfen to increase the impact of autodissemination under semi-field and field conditions [72].

The occurrence of various factors has limited the capability to investigate a broader aspect against the local mosquito population. In addition, incoming mosquitoes may also play an important role in increasing the coverage of pyriproxyfen dissemination, which, unfortunately, may lead to the failure of specific experiments [73]. The oviposition preference of mosquitoes has a complex effect on understanding local populations following the use of a new pyriproxyfen formulation with a potent attractant developed by the manufacturer. Certain studies have reported that *Aedes* spp. express oviposition behavior for certain types of attractants, such as bamboo leaf infusions [74], octenol [75], and yeast-produced CO_2_ [76], and these substrates should be considered when MHAQ is used because of their possibility of enhancing the insecticide transference success rate. Furthermore, further investigation on all aspects of the oviposition substrates that act as important oviposition cues should be performed to improve the MHS efficacy before it can be considered an alternative tool in vector control management.

## Conclusions

The findings in this study demonstrated that MHS has potential for use as an autodissemination station in future dengue control programs. Most of the parameters tested in our laboratory trials showed encouraging results, despite the formulation of the MHAQ showing a weak attractant effect on the *Ae. aegypti.* However, small-scale field trials have shown that MHAQ was more attractive than ovitraps, with more eggs collected. Further research should focus on the identification of the compound responsible for attracting *Aedes* spp. mosquitoes as well as their underlying mechanisms. In addition, it is essential to improve the efficacy of MHS, particularly in semi-field and field conditions, as well as the discovery of new and powerful combinations of MHAQ solutions. The combination of different techniques and approaches may prove to be a powerful alternative tool for vector control programs, particularly in Malaysia.

## Data Availability

Data supporting the conclusions of this article are included within the article. The datasets used and analyzed during the present study are available from the corresponding author upon reasonable request.

## Abbreviations

ANOVA: Analysis of varians
EI: Emergence inhibition
ER: Effective reduction
HI: Hay infusion
MHAQ: Mosquito Home Aqua
MHS: Mosquito Home System
RH: Relative humidity
TW: Tap water
WHO: World Health Organization
